# Development of Carrot Nutraceutical Products as an Alternative Supplement for the Prevention of Nutritional Diseases

**DOI:** 10.3389/fnut.2021.787351

**Published:** 2022-01-03

**Authors:** Nadia Riaz, Zubaida Yousaf, Zarina Yasmin, Muneeb Munawar, Afifa Younas, Madiha Rashid, Arusa Aftab, Bushra Shamsheer, Hamna Yasin, Muhammad Najeebullah, Philipp W. Simon

**Affiliations:** ^1^Department of Botany, Lahore College for Women University, Lahore, Pakistan; ^2^Department of Horticulture, University of Wisconsin-Madison, Madison, WI, United States; ^3^Post-Harvest Research Centre, Ayub Agricultural Research Institute, Faisalabad, Pakistan; ^4^Vegetable Research Institute, Ayub Agricultural Research Institute, Faisalabad, Pakistan; ^5^Department of Botany, Division of Science and Technology, University of Education Lahore, Lahore, Pakistan; ^6^Vegetable Crops Research Unit, US Department of Agriculture—Agricultural Research Service, Madison, WI, United States

**Keywords:** antioxidant, β-carotenoid, genetic diversity, nutraceutical, heritability, morpho-nutritional, physiochemical

## Abstract

Nutraceuticals can serve as an alternative supplement to overcome nutritional deficiency for a healthy lifestyle. They can also play a key role in disease management. To develop carrot nutraceutical products, 64 genotypes from four different continents were evaluated for a range of morpho-nutrition variables. Genetic variability, heritability, strength and direction of association among variables, and direct and indirect relationships among physiochemical and nutritional traits with β-carotene content were evaluated. Core diameter, foliage weight, root weight and shoulder weight showed significant association with β-carotene accumulation. Principal component analysis for physiochemical and nutritional assessment divided these genotypes into two distinctive groups, Eastern carrots and Western carrots. Caloric and moisture content had high positive associations with β-carotene content while carbohydrate content was negatively associated. Five genotypes (T-29, PI 634658, PI 288765, PI 164798, and Ames 25043) with the highest β-carotene contents were selected for making three nutraceutical supplements (carrot-orange juice, carrot jam and carrot candies). These nutraceutical supplements retained high β-carotene content coupled with antioxidant properties. Carrot jam (6.5 mg/100 g) and carrot candies (4.8 mg/100 g) had greater concentrations of β-carotene than carrot-orange juice (1.017 mg/100 g). Carrot jam presented high antioxidant activity with the highest values in T-29 (39% inhibition of oxidation) followed by PI 634658 (37%), PI 164798 (36.5%), Ames 25043 (36%) and PI 288765 (35.5%). These nutraceutical products, with 4–6.5 mg/100 g β-carotene content, had higher values than the USDA recommended dietary intake of 3–6 mg β-carotene/day can be recommended for daily use to lower the risk of chronic disease.

## Introduction

Malnutrition is one of the main health problems emerge widely in the world. Malnutrition and protein-energy coupled with mineral deficiency is among the major causes of global illness and death. While the incidence of extreme hunger has reduced in the last several decades, 821 million people were estimated to be chronically undernourished in 2017 and according to the World Health Organization (WHO), 210 million children and women suffer from vitamin A deficiency (1) and 45% of children below the age of five are malnourished in developing countries with high prevalence in South Asia and Africa (2). Vitamin A deficiency (VAD) is among the main manifestations of malnutrition (3). For example, 51.5% of children in Pakistan are facing VAD with a slightly higher incidence in boys than girls (4). VAD can damage the eye's photoreceptors, leading to vision problems including xerophthalmia. Dietary sources of vitamin A assist in the protection of vision, reduce macular degeneration and suppress the development of senile cataracts, a main cause of blindness (5, 6). Beyond vision impairment, VAD reduces immune function, and it contributes to infertility, morbidity and mortality There are two approaches to overcome these non-communicable diseases (NCDs): prevention and treatment. Prevention can be less expensive than treatment since it involves changing lifestyle habits. For NCDs to be alleviated with dietary changes, researchers are investigating compounds naturally present in nutrient-fortified food including “nutraceuticals” to cure NCDs (7).

Nutraceuticals are described as food or a part of food which can provide health benefits including prevention against chronic diseases (8, 9). Nutraceuticals are indigenous to Indian, Roman, Sumerian and Chinese civilizations (10). Frequent consumption of nutraceuticals is very common in people of every age. In the USA ~ 55% of children often consume nutraceuticals as vitamins and mineral supplements (11). The use of dietary supplements has increased over the past 20 years. Hence, dietary supplements comprise an estimated ~!$30 billion in the United States and ~$100 billion globally (12, 13). In 2020, the U.S dietary supplements sale has significantly increased by 255% and 415% due to the pandemic COVID-19 (14). Nutraceuticals range from isolated nutrients to complex dietary supplements. Probiotics, prebiotics, dietary fibers, fatty acids and antioxidants are all categorized as dietary supplements (15).

Human beings and all animals are ultimately dependent on carotenoid pigments in plants to supply their vitamin A needs. A subset of the 600 to 800 carotenoids includes provitamin A carotenoids such as α-carotenes, β-carotenes (16), different xanthophyll like zeaxanthin, neoxanthin, violaxanthin, lutein (Sakar and Oba, 2020) and β-cryptoxanthin which, when consumed by animals, are converted to retinol (17). Carotenes and antioxidants (18) with anti-cancer, anti-cataract, anti-urinary tract infection, lowered blood pressure and reduced muscular degeneration properties (10, 17). Vegetables are an inexpensive source of carotenoids and carrot is one of the few plants of the family Apiaceae with massive carotenoid levels occurring in storage roots that have been estimated to provide 67% of the α-carotene and 28% of the β-carotene in the US diet (19) and 60–82% of the α-carotene and 60–90% of the β-carotene in several European diets (20).

Carrot (*Daucus carota* L.) is an economically important crop with diverse range of phenotypic and genotypic variation and with global production having been adapted for production in Europe, Asia and the Americas (21). Carrot is a biennial crop, having favorable cultivation from September to November in tropical and subtropical regions whereas, temperate regions can have an extended cultivation period throughout the year (22). Globally, carrot production has risen progressively in the last 50 years (23), with a three-fold increase in production area (383,965–1,166,885 ha) and two-fold increase in yield (166,893–329,021 hg/ha) to result in a six-fold increase in total production. With these increases, the average global increase in per capita carrot production has risen 2.7-fold in the last 50 years (24).

The domestication of carrot included increased carotenoid, anthocyanin and sugar content, loss of lateral root branching, biennial growth habit, and increased size and variation of root shape (25). Carrot colors include white, orange, yellow, red, and purple with each color comprising nutritionally valuable phytochemicals including carotenoids, anthocyanins, and other phenolic compounds. This makes the vegetable a good source of dietary antioxidants (26). The most abundant antioxidant compounds found in carrots are α- and β-carotene, vitamin E, and anthocyanin. Interestingly, the levels of these antioxidant pigments found in different cultivars are responsible for the colors of carrots. The carotenoids α- and β-carotene, lycopene, and lutein account for the orange, red, and yellow colors, respectively (27). Orange-colored carrots are unusual for their high α-carotene fraction ranging from 13 to 40% of their total carotenoids, with β-carotene accounting for most of the rest. Red carrots always contain lycopene and usually also contain α- and β-carotene along with lutein (26, 28, 29). Besides high bioavailability of carotenoids, carrots have also a unique combination of three flavonoids: kaempferol, quercetin and luteolin (30) and other phenolic derivatives stimulating cancer-fighting mechanisms in the human body (31). Carrot is also a good source of dietary fibers and trace mineral elements. Molybdenum, magnesium and manganese found in carrots help in carbohydrate metabolism, energy production, absorption of iron (32), insulin secretion (33, 34) and coordination of antioxidant enzymes in the body whereas, potassium helps in functioning of muscles (5, 6). Carrot extracts have been reported in experimental studies to possess cardio- and hepatoprotective (35–37), to reduce Alzheimer's and other dementia disorders (38–40), and to provide anti-bacterial and anti-fungal properties (41–43), anti-inflammatory and analgesic benefits (38, 44) and fertility benefits.

As carrot contributes 28–90% of β-carotene taken by humans (19, 20), several attempts have been made to utilize carrot in the form of its value-added raw, cooked or processed products (45). Carrot juice enriched with α- and β-carotenes has become the regular part of diet by some people throughout the world possessing high vitamin C content (46). Concomitant with the variation in carrot color, nutrient composition in diverse germplasm helps to characterize cultivars where darker color is typically associated with higher nutritional value (19, 47). Moreover, information on nutritional properties of genotypes is very important to compliment basic phenotypic and genetic characterization. So far, no such work has been done in Pakistan to develop new carrot products. As market-based nutraceuticals and nutrient-fortified foods are quite expensive and out of the reach of the poor people of developing countries, this project was designed to develop carrot products in the form of carrot-orange mix juice, carrot candies and carrot jam as a nutritionally acceptable snacks for poor communities, especially children. This project also provides nutraceutical products useful to further explore nutrient bioavailability in clinical studies.

## Materials and Methods

The current project was planned in the Department of Botany, Lahore College for Women University, Lahore and executed in collaboration with Vegetable Research Institute and Post-Harvest Center, Ayub Agricultural Research Institute Faisalabad, Pakistan (48).

### Plant Material

A panel of 64 genotypes consisting of 62 cultivated and 02 wild genotypes was obtained from Genetic Resource Information Network, United States Department of Agriculture, USA representing carrots from seventeen (17) countries of Asia, North America, Africa and Europe.

### Field Experiment

Field trials were carried out in fall 2018, 2019, and 2020. The experiment was arranged in a randomized complete block design with three replications. The soil was sandy loam that was prepared by three ploughings followed by three finer cultivations to break up soil clods and the soil was leveled. Seed was treated with Imidacloprid WS 70% at 3 g kg^−1^ before sowing. Individual plot size was 5.0 × 2.5 m with inter-row distance of 40 cm. Nitrogen (N) from urea, phosphorus (P) from P_2_O_5_, and potassium (K) from muriate of potash were applied at 150:85:60 kg ha^−1^. All the P and K and 1/3 of the N were applied before sowing. Successive irrigations were applied depending upon weather conditions to keep soil at field capacity. During this experiment F_1_ and F_2_ progenies seed was developed by selfing and roots were further used for phenotypic, nutritional evaluation and development of nutraceutical products. Roots were harvested 90–100 d after planting and stored at 4°C until analysis.

### Characterization of Important Morpho-Nutritive Parameters

Morphological parameters add color characteristics related to nutrient content of carrots were evaluated in F_1_ and F_2_ generations. Root weight (g), shoot weight (g), root diameter (mm), root shoulder width (mm), petiole thickness (mm), lateral root growth, root shape, green color on shoulder, red color on shoulder, root tapering, root tip shape, root surface pigmentation, core color and cortex color were noted according to IPGRI (49) descriptor of wild and cultivated carrots. Passport data of 64 carrot genotypes along with recorded morpho-nutritive parameters is also provided (Supplementary Table 1).

### Evaluation of Physiochemical and Nutritional Parameters

#### Sample Collection and Preparation

Mature carrots of different colors from F_2_ generation were sent to the Post Harvest Institute for nutritional profiling based upon 2 years phenotypic screening. Selected roots of 64 genotypes were thoroughly washed, trimmed and air dried to make a fine powder. This powdered material was further used for testing proximate analysis (50), root color analysis and β-carotene.

#### Determination of Total Soluble Solids

Total soluble solids were determined through hand refractometer with automatic temperature compensation and the values were expressed as degrees Brix.

#### Determination of pH

pH of the carrot juices was determined with a pH meter and display reading was noted.

#### Determination of Acidity

Acidity was estimated through titration against 0.1 N NaOH in the presence of phenolphthalein indicator until reaching the light pink color end point.

#### Determination of Vitamin C Content

A titrimetric method estimation was performed by using 2–6 dichlorophenolindophenol dye for samples based on comparison to standard value.

#### Determination of Moisture Contents

2.0 g of root ground sample was dried in crucibles at 105°C for 72 h. The difference in weight was estimated as the moisture content.

#### Determination of Ash Contents

2.0 g samples were placed in crucibles and ashed at 500°C for 3 h to burn organic matter leaving inorganic residue. Ash was cooled in a desiccator and weighed to measure weight loss.

#### Determination of Crude Fats

Crude fats were determined by the Soxhlet extraction method. Five grams moisture sample was put into a thimble along with 150 mL n-hexane as solvent for the extraction and heated at 60°C for 2 h under reflux on a heating mantle. The extract was then air dried at 100°C, cooled and weighed for fats estimation.

#### Determination of Crude Fibers

Approximately 2.0 g of the sample was poured into a volumetric flask (1 L preheated) followed by the addition of 150 mL, 0.128 M H_2_SO_4_. The solution was refluxed for 30 min and cooled to room temperature. This was filtered through an ashless filter paper, and the residue washed with hot water (10 mL × 3). Exactly 150 mL of preheated 0.22 M KOH was added to the residue and refluxed for 30 min. It was cooled, filtered, and the residue was washed with acetone. The residue was oven-dried at 130°C for an h, weighed, and the dried residue was ashed at 500°C for 3 h cooled and weighed. The loss in weight was used to estimate fiber content.

#### Determination of Protein Contents

Crude protein contents was determined by the micro Kjeldahl method (51). Protein content was estimated using the relationship: % protein content = *N* × 6.25, where *N* is the nitrogen content, and 6.25 is the protein conversion factor. Approximately 1.0 g carrot powder of each variety was poured into a Kjeldahl digestion flask and followed by the addition of a catalyst (2.0 g of potassium sulfate, 1.0 g of copper sulfate and 0.1 g selenium powder) and 10 mL concentrated H_2_SO_4_. The flask was heated continuously in a fume hood until a green solution was obtained. The heating continued for about 30 min before cooling. Distilled water (10 mL) was added to the cooled digest and vigorously shaken. The digest was transferred into 100 mL volumetric flask and topped up to the mark with water. A mixture of 10 mL aliquot of the digest and 10 mL of 40% NaOH was distilled for 5 min into a receiver containing boric acid (10 mL, 2% w/v) using a Markham distillation unit. The distillate was titrated with 0.01 M HCl to determine the nitrogen content. Percent of crude protein was calculated by multiplying percent of Kjeldahl nitrogen with 6.25.

#### Determination of Carbohydrates

Percentage of carbohydrates was estimated by following formula:


$$\begin{array}{l} \%\text{Carbohydrates}=100(\%\text{ash}+\%\text{moisture}+\%\text{fat}+\%\text{fiber }\\ \;+\%\text{protein}) \end{array}$$

#### Determination of Calories

Calories were estimated using the following formula.


$$\begin{array}{l} \text{Calories}=(\%\text{protein}\times 4)+(\%\text{fat}\times 9)\;\\ \;+(\%\text{carbohydrates}\times 4) \end{array}$$

#### Determination of Color

Color properties of selected carrot roots were measured using a spectrophotometer. The color of the specimen was measured with a spectrophotometer (SP820λ; Techkon Gmbh, Konig-Stein, Germany) against a black background to stimulate the absence of light. All samples were chromatically measured in triplicate and each color parameter was averaged. The CIE (1976) L^*^a^*^b^*^ color system was used for the determination of color. The measured parameters were L^*^ (lightness/darkness), a^*^ (red/green) and b^*^ (yellow/blue). Then color was calculated by the below given formula:


$$\begin{array}{l} \text{ΔEab}*={\left[{\left(\text{ΔL}*\right)}^{2}+{\left(\text{Δa}*\right)}^{2}+{\left(\text{Δb}*\right)}^{2}\right]}^{1/2} \end{array}$$

Whereas, L^*^ is for lightness from white to black, a^*^ red/green (a = green, +a = red), b^*^ is blue /yellow (-b = blue, +b = yellow).

### Analysis of Carotenoids

#### Carotenoid Extraction

Carotene extracted from carrots for “Reversed phased HPLC system” by the method of Khalil and Varananis (52). Ten grams of sample was homogenized in 30 mL of acetone. 0.1% (BHT) solution in acetone was added as an antioxidant. The resulting extract was filtered through Buchnar's funnel. The residue was washed twice with acetone until it become colorless. The filtrate was combined with 20 g of anhydrous sodium sulfate and anhydrous sodium sulfate removed through filtration and the volume of extract reduced by rotatory evaporator. The extract was transferred quantitatively to 100 mL volumetric flask and the volume was made up to the mark with acetone and water, so that the final extract contained 80% of acetone. Standard of β-carotene was supplied by Sigma-Aldrich (Germany). Stock solution of β-carotene was prepared dissolving 100 mg in 100 mL n-hexane. Chromatographic was analysis was performed with a Perkin Elmer HPLC programme using a LC-1000 pump (Isocratic), C18 column and LC 250 UV/VIS detector was used. Peak identification and quantification were made by “CSW 32 software” for the HPLC system. The HPLC was calibrated by running mobile phase (acetonitrile, dichloromethane and methanol volume ratio of 70:20:10, respectively) at a rate of 2 mL per min. Wavelength was fixed at 452 nm. The pressure of the column was kept 1800-2000 PSI. Each standard solution (20 μL) of β-carotene was injected when the injector was in load mode. The standard β-carotene peak was achieved at the retention time of 4.7 min (Rt = 4.7). The concentrations of the β-carotene standards were plotted against the peak area to obtain a straight line.

#### Sample Assay

Each sample of carotenoid extract in 80% acetone was used for HPLC assay like the standard; each carrot sample (20 μL) was taken by micro liter syringe. The peak was automatically identified and quantified.

### Antioxidant Activity by DPPH Scavenging Assay

#### Sample Preparation and Analysis

Antioxidant capacity of carrot products was evaluated monthly up to 3 months. DPPH method (26) was used for the determination of antioxidant capacity of biofortified carrots was followed. The products were dried to make powder. Aliquot of extracted products were added to 1 mL of DDPH in ethanol solution and kept at room temperature for 30 min. After that, the absorbance was determined at 517 nm on spectrophotometer. The reduction of the absorbance was calculated according to the following equation:


$$\begin{array}{l} \text{Inhibition}\%=\left[{\text{Abs}}_{\text{t}=0}-{\text{Abs}}_{\text{t}=30\text{min}}\right)/{\text{Abs}}_{\text{t}=0}\times 100 \end{array}$$

Where Abs_t = 0min_ and Abs_t = 30min_ were the absorbance of DPPH solution at 0 and 30 min, respectively. The calculated inhibition percentage was used to express antioxidant capacity. The reduction of the absorbance against the amount of sample. Butylated hydroxytoluene (BHT) and α-tocopherol were used as a standard.

### Development of Carrot Products

#### Sample Collection and Preparation

Five genotypes were selected for the development of carrot products. The selection criteria were based upon proximate evaluation. After washing and trimming, selected carrot roots were utilized to make juice, candies and jam. The product shelf life was evaluated monthly for 90–100 d with further evaluation of TSS, pH, acidity, vitamin C, color values, mineral matter content, dry matter content, carotenoid, and antioxidant activities to safeguard quality assurance. Occurrence of spoilage was noted.

#### Carrot Mix Juice

Juice was extracted by using a local juice blender. Market purchased fresh oranges were washed. Orange juice was extracted and filtered with muslin cloth to remove large fiber particles. Carrot juice was extracted by using juice extractor and 50% carrot- 50% orange juice was prepared according to the standard formulation methods of (50) which includes addition of sodium benzoate to prevent spoilage, carboxymethyl cellulose (CMC) powder for viscosity enhancement and citric acid to stabilize carrot and orange concentration as preservatives and stored at room temperature (±27°C) in the dark.

#### Carrot Candies

Carrots were blanched for 5–7 min then cooled. Sugar syrup of 30 brix was used for osmo-dehydration. Roots were dipped in the sugar syrup for 24 h, liquid drained, and carrots cut into round chewable pieces. A tunnel dehydrator was used to dry the pieces up to 30–35% moisture content. The samples were packed in transparent ziploc bags to make it visible for any contamination and stored in the dark at room temperature (±27°C).

#### Carrot Jam

One kg peeled carrot was cut into small chunks and dipped in 8% salt solution to maintain its color consistency. After 2 h plain water was added into the salt solution and the mixture was boiled for five min for blending. After fine blending 500 g sugar and 2 g citric acid was added and the mixture cooked until thickening. The cooked jam was removed from heat and 1 g sodium benzoate was added. After cooling down to 85°C jam was transferred into glass jars for storage in a dry place at room temperature.

### Data Analysis

The nutritional data for 64 genotypes was assessed for variance analysis (53) and significance was tested at *p* < 0.05, *p* < 0.01, and *p* < 0.001 *via* F-test.

The broad sense heritability (h^2^b) was calculated as ${\text{σ}}_{\text{g}}^{2}$/${\text{σ}}_{\text{p}}^{2}$ (54). The heritability was classified as low (<50%), medium (50–80%) and high (>80%) (55, 56).

The genetic advance was computed as, GA = h^2^b × σ_p_ × K, where σ_p_ and K represent phenotypic standard deviation and standardized selection differential constant) at 5% selection intensity, respectively (57). The GCV (genotypic coefficient of variation) and PCV (phenotypic coefficient of variation) were computed by Ogunniyan and Olakojo (57) and heat map was constructed for respective comparison.


$$\begin{array}{l} \text{PCV}\left(\%\right)={\text{σ}}_{\text{p}}/\bar{\text{x}}\times 100\\ \text{GCV}\left(\%\right)={\text{σ}}_{\text{g}}/\bar{\text{x}}\times 100 \end{array}$$

σ_p_ = phenotypic standard deviation σ_g_ = genotypic standard deviation.

rp = phenotypic correlation and rg = genotypic correlation was calculated following the formula of Sarker et al. (58), Sarker et al. (59), and Sarker et al. (60). Path coefficient analysis was performed following the formula of Sarker et al. (58), Sarker et al. (59), Keles et al. (61) to evaluate the direct and indirect effects of the most highly correlated nutrients on β-carotene content.

To measure variation, minimize the dimensionality of variables and to assess the contribution of each genotype with phenotypic and nutritional parameters, principal component analysis was performed (62). The PCA was generated by using R statistical package “ggbiplot” in R studio version 2020. The K-means clustering algorithm was used as per following function to define k centroids, one for each cluster (63). Here, k is the number of cluster centers, n is number of data points from respective cluster center. The data was subjected to default K means function.


$$\begin{array}{l} J=\sum {}_{j=1}^{k}\sum {}_{i=1}^{n}\|x^{2}{}_{\text{i-j}}^{\text{i c}}\| \end{array}$$

where J is objective function, k is number of clusters predefined and n is number of case. $\left\|x^{2}{}_{\text{i-j}}^{\text{i c}}\right\|$ is distance function of Euclidean distance, x^i^ is case, i and c_j_ is centroid for cluster j.

Further Silhouette plot analysis function was carried out in the R programming software (R Studio 2020) for validation of consistency within clusters of data and to determine how well an observation is clustered. Cluster analysis Silhouette coefficient was calculated by using the following formula:


$$\begin{array}{l} {\text{S}}_{\left(\text{i}\right)}={\text{b}}_{\text{i}}-{\text{a}}_{\text{i}}/\text{max}\left({\text{a}}_{\text{i}},\;{\text{b}}_{\text{i}}\right) \end{array}$$

The S_i_ varies between −1 ≤ Si ≤ 1

All tests and analyses for carrot products were performed in triplicate with sample preparation and handling being performed twice, and the obtained data were averaged. The differences between mean and standard error were calculated by SPSS 16.0 statistical software.

## Results

### Variation and Genetic Parameters of Variability, Heritability, and Genetic Advance for Nutrition

Genotype of carrots evaluated affected all the physiochemical and nutritional parameter (Table 1). Considerable variation existed among the genotypes able to be exploited for nutritional crop breeding was used to select more nutritious carrots for product development. Environmental variation was relatively small but slightly higher for moisture, calories, carbohydrates, and carotene. This variation was also confirmed by those same values as measured in the analysis of genotypic coefficient of variation, phenotypic coefficient of variation and broad sense heritability. These results indicate that genetic variation plays a more important role in expression of these traits than environment. Important for this study, genetic variation for β-carotene was found to be slightly higher than phenotypic variation. The GCV depicted true genetic potential of genotypes under study. High genetic advance values were observed for vitamin C, color, moisture, calories, and carbohydrates.

**Table 1:** ANOVA and genetic parameters of variability for fifteen (15) important nutritional traits of 64 *Daucus carota* L. genotypes.

**Characters**	**TSS**	**pH**	**Acidity**	**Vit. C**	L*	a*	b*	**Moisture**	**Ash**	**Crude fat**	**Crude protein**	**Crude fiber**	**Calories**	**Carbs**	**β-carotene μg/100 g**
DF	63	63	63	63	63	63	63	63	63	63	63	63	63	63	63
Mean square	12.413	0.473	0.273	474.42	295.23	26.670	210.609	2,089.99	0.0174	0.0067	0.016	0.020	2,102.65	2,095.59	444,559
sum square	782.05	29.761	17.173	29,888.4	18,599.3	53.3	13,268.4	131,670	1.093	0.42542	1.039	1.252	132,47	132,022	280,071,654
S.E of Mean	0.059	0.034	0.009	0.345	0.277	0.172	0.233	0.732	0.003	0.003	0.003	0.002	0.732	0.767	33.757
σ^2^e	0.010	0.004	0.0002	0.356	0.230	0.088	0.162	1.605	0	0	0	0	1.608	1.615	3,418.625
${\text{σ}}_{\text{g}}^{2}$	4.134	0.156	0.0908	158.020	98.330	37.380	70.150	696.130	0.006	0.002	0.005	0.007	697.993	700.347	1,480,721.063
${\text{σ}}_{\text{p}}^{2}$	4.145	0.1598	0.091	158.377	98.562	37.466	70.311	697.735	0.006	0.002	0.005	0.007	699.601	701	1,484,139.689
GCV	29.480	6.0366	19.782	61.833	24.100	45.565	42.188	34.676	6.971	15.143	7.382	6.864	123.532	137.843	21.422
PCV	29.516	6.104	19.804	61.903	24.128	45.619	42.233	34.716	6.971	15.143	7.382	6.864	123.674	138.003	21.447
h^2^b	0.997	0.9781	0.998	0.998	0.998	0.998	0.998	0.998	1	1	1	1	0.998	0.998	0.998
GA	4.183	0.805	0.620	25.866	20.404	12.579	17.234	54.289	0.156	0.097	0.153	0.167	54.362	54.453	2,503.819
GA % of mean	30.652	12.297	40.709	127.232	49.588	93.753	86.808	71.351	14.362	31.188	15.210	14.143	254.183	283.631	44.079

*DF, degree of freedom; S.E, standard error; σ^2^e, Environmental variance; $\sigma_{\text{p}}^{2}$, phenotypic variance; $\sigma_{\text{g}}^{2}$, genotypic variance; PCV, phenotypic coefficient of variation; GCV, genotypic coefficient of variance; h^2^b, heritability broad sense; GA, genetic advance at 5% selection. Phenotypic and genotypic variation values were significant at the 95% value for all traits*.

### Correlation Analysis

**Figure 1:**
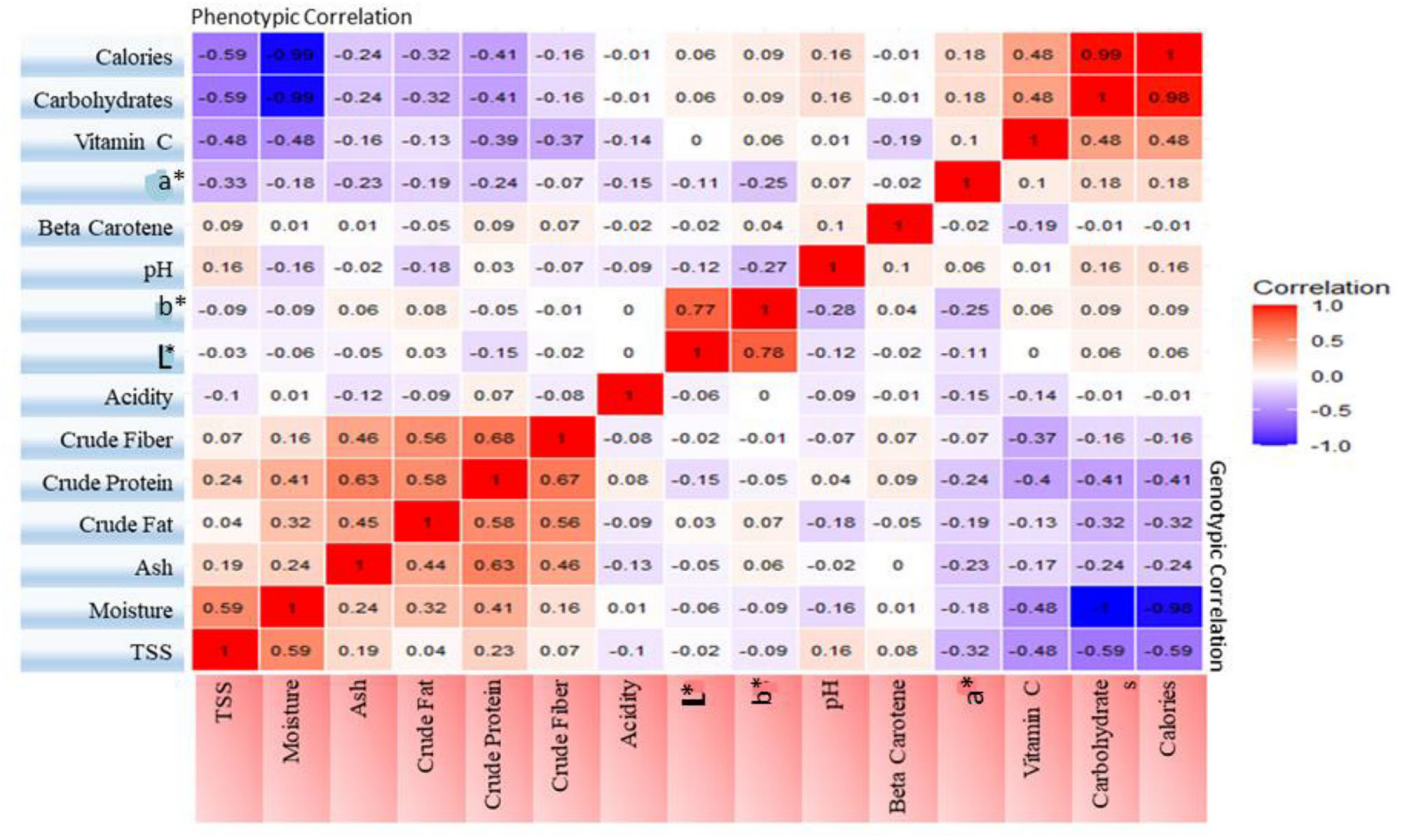
Heat map showing phenotypic and genotypic correlation coefficients among fifteen nutritional characters.

A heat map of genotypic and phenotypic correlation coefficients was constructed among the nutritional parameters (Figure 1). The genotypic correlation coefficients were very closely correlated to the phenotypic correlation coefficients. Among genotypic correlation coefficients, TSS and moisture (0.59) were positively correlated, and both factors were negatively correlated with CIE color parameter a^*^ (−0.33, −0.32), vitamin C (−0.48), carbohydrates (−0.59) and calories (−0.59). The latter three factors were highly correlated among each other. Crude protein was significantly correlated with moisture (0.41), ash (0.63), crude fat (0.58) and crude fiber (0.68) while moisture was negatively correlated with carbohydrates (−1) and calories (0.98). β-carotene was found to be non-significant with all parameters for both correlation coefficients.

### Genotypic Path Analysis

**Figure 2:**
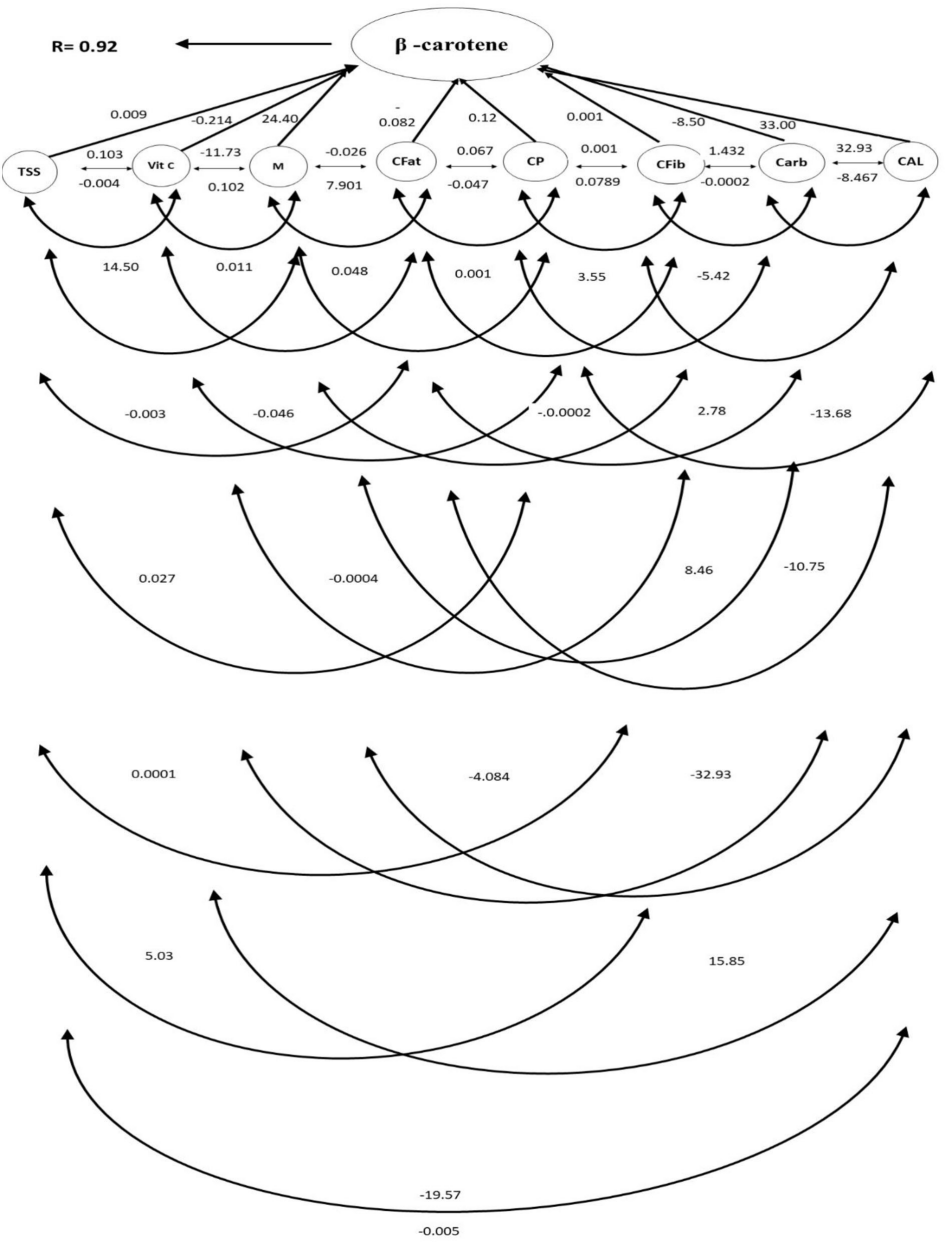
Path diagram showing direct and indirect effects of eight significantly important nutritional characters of 64 *Daucus carota* L. genotypes on β-carotene content. TSS, total soluble solids; Vit. C, vitamin C; M, moisture; CFat, crude fat; CP, crude protein; Cfib, crude fiber; Carb, carbohydrates; Cal, calories; R, residual effects.

Genotypic path analysis provided an evaluation of direct and indirect effects of the eight most highly correlated proximate parameters on β-carotene content (Figure 2; Table 2). In a similar pattern observed with those correlation values, β-carotene content was most directly associated with calories (33.0), moisture content (24.4) followed distantly by crude protein (0.12). vitamin C (−0.214), carbohydrates (−8.5) and crude fat (−0.12) showed negative direct effects. TSS affected β-carotene positively through vitamin C, moisture, crude protein, and carbohydrates except for its non-significant direct effect. Vitamin C had negative effects with all parameters except for calories. Moisture interacted negatively with crude fat, crude fiber, and calories while vitamin C and crude fat increased with β-carotene content and with vitamin C, crude protein, and calories. Crude fat interacted positively with vitamin C, moisture, crude protein, and carbohydrates, while negatively with calories. Crude protein interacted positively with vitamin C, moisture, and carbohydrates while negatively with calories and fat. Crude fiber had a positive relationship with vitamin C, moisture, crude fat, protein, and carbohydrates. TSS negatively interacted with β-carotene, carbohydrates, and calories (Table 2).

**Table 2:** Estimation of direct and (bold numbers) indirect effects of nutritional parameters of 64 *Daucus carota* L. genotypes on β-carotene content.

**Character**	**TSS**	**Vitamin C**	**Moisture**	**Crude fat**	**Crude protein**	**Crude fiber**	**Carbohydrate**	**Calories**	**β-carotene**
TSS	**0.009**	0.103	14.50	−0.003	0.027	0.0001	5.03	−19.57	0.089
Vitamin C	−0.004	**−0.214**	−11.73	0.011	−0.046	−0.0004	−4.084	15.85	−0.228
Moisture	0.005	0.102	**24.40**	−0.026	0.048	−0.0002	8.46	−32.93	0.052
Crude fat	0.0004	0.029	7.901	**−0.082**	0.067	0.001	2.78	−10.74	−0.045
Crude Protein	0.002	0.085	10.078	−0.047	**0.12**	0.001	3.55	−13.68	0.104
Crude Fiber	0.0007	0.079	3.964	−0.046	0.0789	**0.001**	1.432	−5.42	0.092
Carbohydrates	−0.005	−0.103	−24.395	0.027	−0.049	−0.0002	**−8.50**	32.93	−0.053
Calories	−0.005	−0.103	−24.396	0.026	−0.048	−0.0002	−8.464	**33.00**	−0.052

### Principal Component Analysis of Morpho-Nutritional Parameters

**Figure 3:**
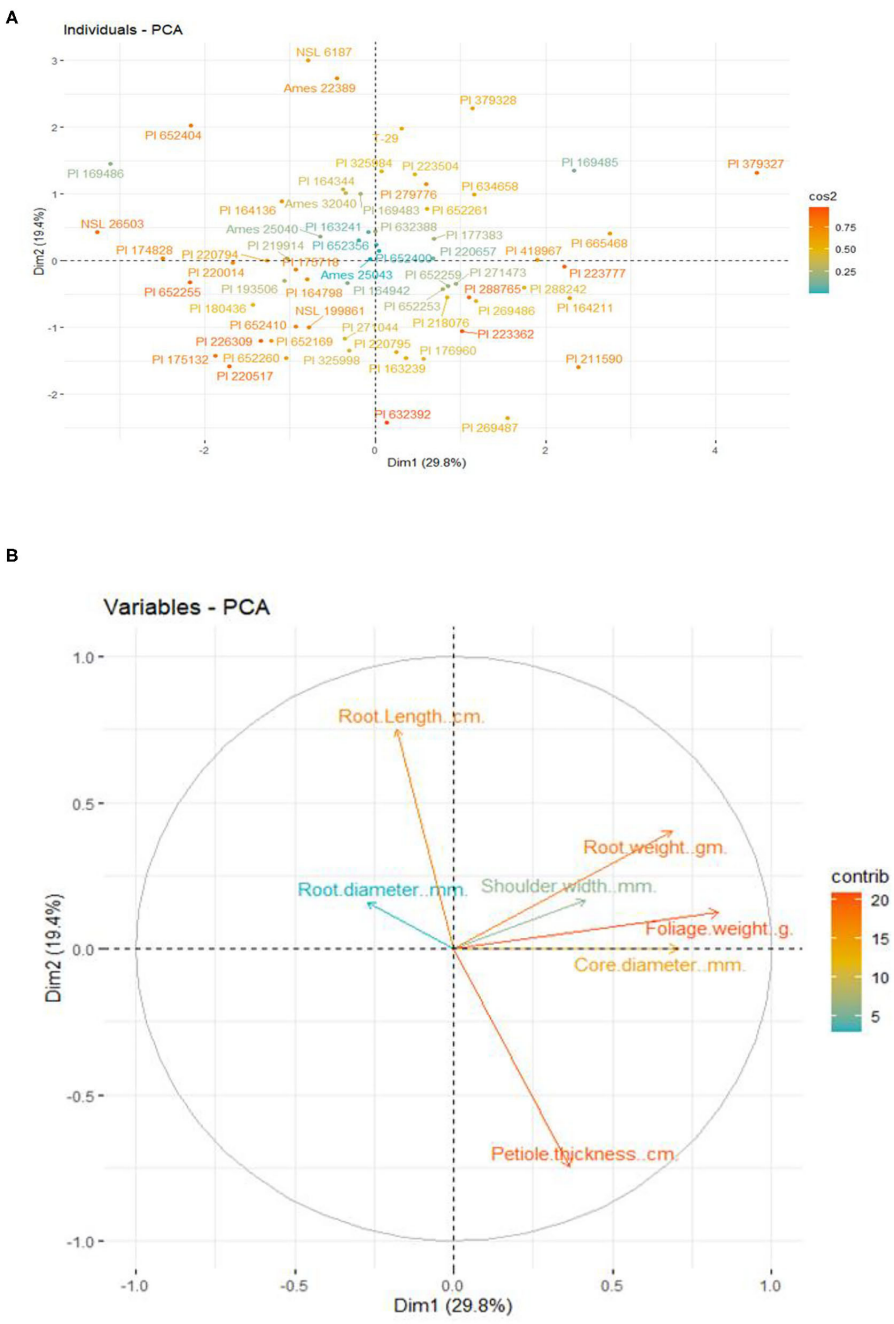
**(A)** Classification of 64 *Daucus carota* L. genotypes for morpho-nutritional parameters based on the principal component analysis **(B)**. PCA analysis depicting the relationship between variables and their contribution in total variation of carrot morphological traits.

Principle component analysis (PCA) was used to evaluate the relationships of individual carrot genotypes with quantitative morphological parameters associated with yield and market quality (Figures 3A,B). Principal components 1 and 2 (PC1 and PC2) had eigenvalues of 2.084 and 1.361, respectively, with a combined variance percentage of 49.2 (Table 3). PCA analysis demonstrated the maximum variation in genotypes belonging to Turkey, Afghanistan, Pakistan and the United States for core diameter, foliage weight, root weight, and petiole thickness parameters (Figure 3B). Petiole thickness, root and foliage weight were most significantly correlated, accounting for 20% of the variation while, root diameter was negatively significant with least contribution (Figure 3B). Root length was strongly negatively correlated with petiole thickness. Foliage weight revealed no significant relationship with root length. Five genotypes (T29, PI 164798, PI 634658, PI 288765, and Ames 25043) were considered the best portraying considerable root characteristics based upon root color, root length, core size and sweetness which contribute to the yield enhancement, consumer attraction and genetic diversity.

**Table 3:** Principal component analysis of quantitative traits of 64 *Daucus carota* L. genotypes.

**Parameters**	**PC1**	**PC2**	**PC3**	**PC4**
Eigenvalue	2.084	1.361	0.986	0.908
Variance percentage	29.8	19.4	14.087	12.976
Cumulative variance percentage	29.784	49.158	63.245	76.221

**Figure 4:**
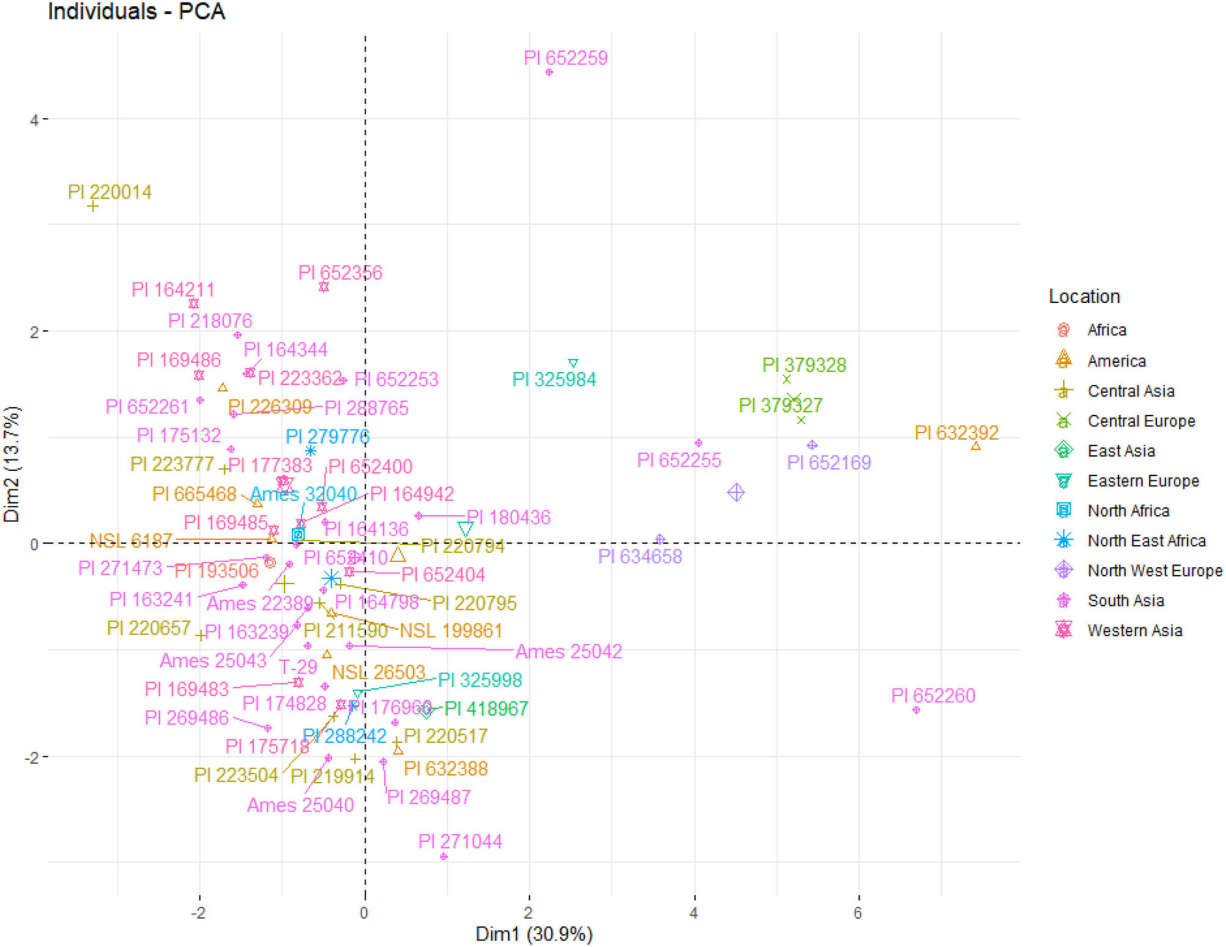
Principal component analysis of 64 carrot genotypes from 17 countries based on nutritional parameters.

Genotypes from Russia, Serbia, and France were closely associated based upon nutritional character profiles measured in this study (Figure 4). While one genotype belonging to Ukraine was positioned in the Asian group. Similarly, many American genotypes were associated with Asia, which is carrot's center of origin. PCA biplot showed the most significant positive associations with pH, vitamin C, carbohydrates, calories, and color. In contrast, crude fat, crude protein, moisture, TSS and ash were most negatively correlated. β-carotene had the minimal variation among genotypes.

**Figure 5:**
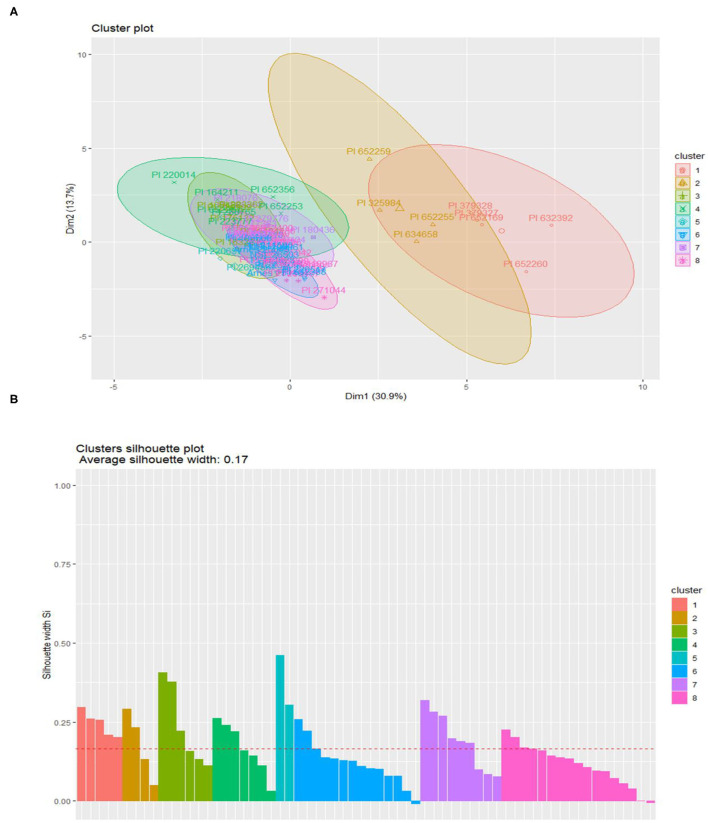
**(A)** K-means clustering of 64 carrot genotypes grouped into 8 k-clusters represented by different colors based upon nutritional profiling. **(B)** Validation of k-means clusters by Silhouette plots. The outliers are in blue and pink color.

The K-means cluster results were validated with portraying Silhouette plots to determine the similarity of genotypes within each cluster and differences with other clusters (Figure 5A). The silhouette coefficient width (Si) grouped these genotypes into 7 clusters. The five genotypes selected for nutraceutical products development, PI 164798, PI 634685, PI 288765, Ames 25043, and T29, were positioned in clusters 5, 7, and 8, respectively. The Si ranged in variation from 0.11 to 0.38. Cluster 5 had the highest Si average width (38), medium with cluster 7 (0.19) and the lowest with cluster 8 (0.11). Si average width was directly proportional to the genetic diversity among genotypes (Figure 5B).

### Proximate Composition and Antioxidant Activity of Carrot Nutraceutical Products

**Figure 6:**
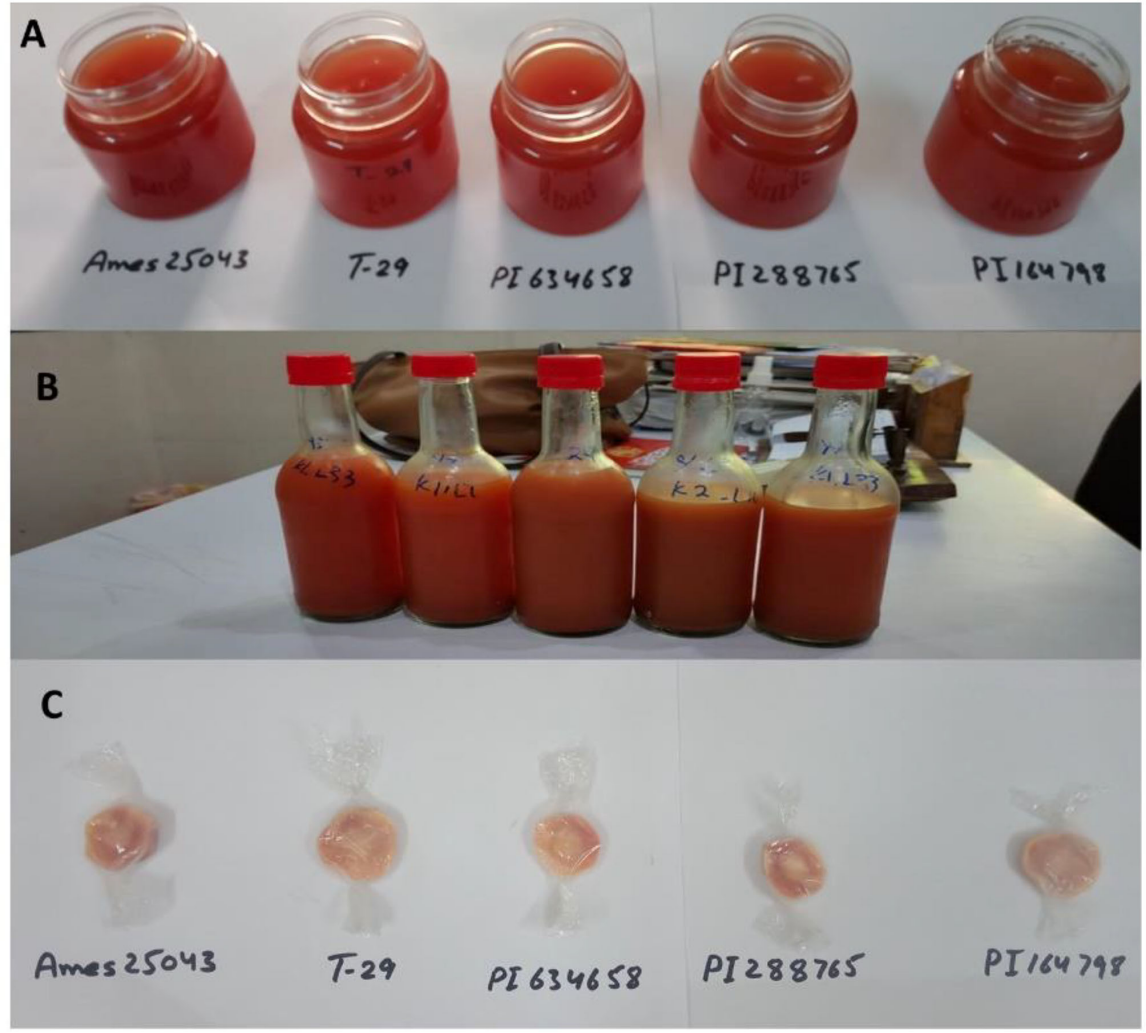
Carrot nutraceutical products i.e., **(A)** Carrot Jam, **(B)** Carrot mix juice, and **(C)** Carrot candies.

Carrot candies, carrot juice and carrot-orange mix juice were prepared (Figure 6) and their proximate analyses for TSS, moisture, crude fat, crude fiber, and protein varied significantly. In the present study mineral content, dry matter, β-carotene and antioxidant activities were evaluated for 3 months (March, April and May) for products prepared in February. Variation among genotypes and variation during storage were both small for all products.

**Figure 7:**
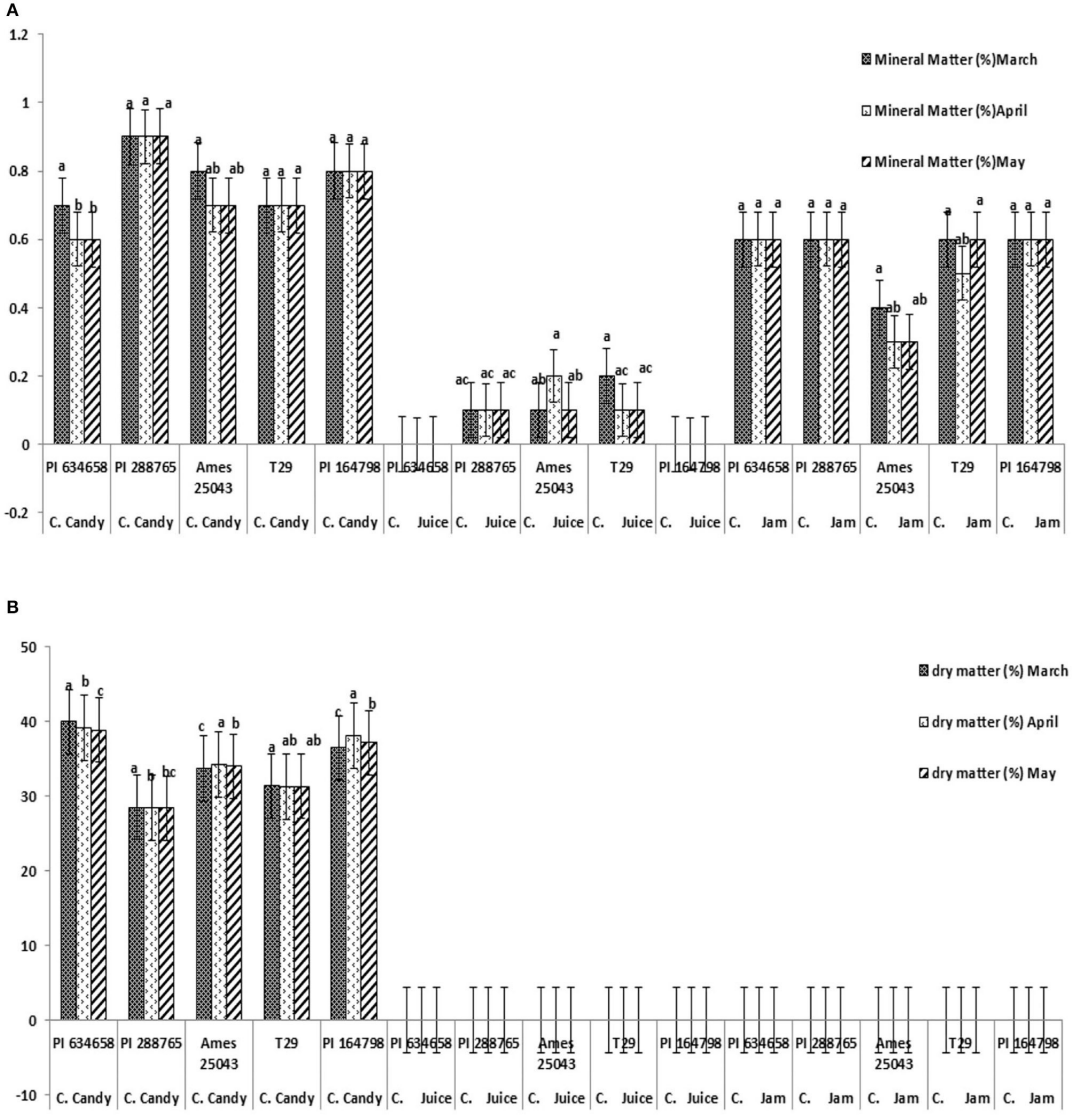
**(A)** Mineral content (%) of three nutraceutical products from five carrot genotypes stored 3 months. **(B)** Dry matter content (%) of three products from five carrot genotypes stored 3 months.

**Figure 8:**
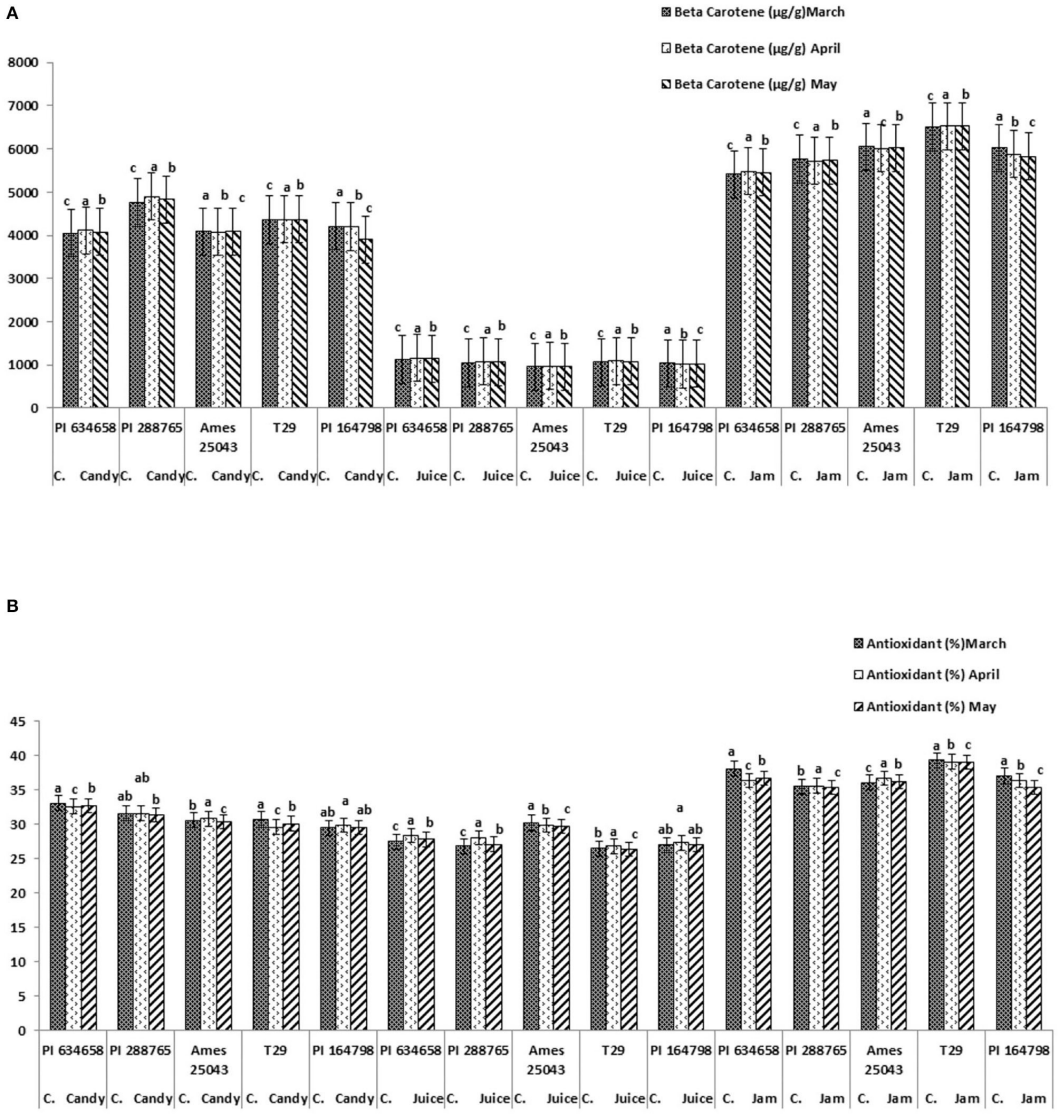
**(A)** β-carotene content (μg/100 g) of three nutraceutical products from five carrot genotypes stored 3 months. **(B)** Antioxidant activity (%) of three products from five carrot genotypes stored 3 months.

The highest value of mineral content was recorded for candies (especially PI 288765 and PI 164798) followed by jam similar for all genotypes except lower for Ames 25043). and least in mix juice (PI 634658 and PI 164798) (Figure 7A). Dry matter was calculated only for candies where, PI 634658 and PI 164798 products had the most promising results followed by Ames 25043, T-29 and PI 288765 (Figure 7B). β-carotene content was higher in all genotypes when used for jam with the highest in T-29 (6.5 mg/100 g) followed by Ames 25043 (6.0 mg/100 g), PI 164798 (5.6 mg/100 g), PI 288765 (5.5 mg/100 g), and PI 634658 (5.4 mg/100 g) (Figure 8A). Carotene content in candies was lower than in jams and was much lower in juice. Similar antioxidant values were observed in all genotypes and products ranging from 27 to 39% (Figure 8B). The highest antioxidant values were observed in T29 carrot jam.

## Discussion

Plants are essential source of secondary metabolites, such as phenolic and flavonoids including hydroxycinnamic acids (64), hydroxybenzoic acids (65), flavones (66), flavanols, flavanones (66), tocopherols (67), betalain (16), ascorbic acids (67), carotenoids (27), betacyanin, betaxanthin (68), chlorophyll a (69), chlorophyll b (70) etc. that have high radical quenching ability (71). These secondary metabolites and some antioxidant enzymes associated with some physiological activities, such as reduce reactive oxygen species (ROS) (72), osmotic stress (18), oxidative damage (73), decrease in photosynthetic activities (71), improve nutrient imbalance (71), in plant cells, protect plants from drastic reduction in growth and productivity (74) and ultimately enhance the concentration of antioxidants (75) that can be used in human diet. The properties of some plants used historically as medicines have been investigated but relatively little has been reported for carrot. Before domestication, carrot seed was used as a traditional medicine in Iranian culture for healing gynecological disorders (76). With the discovery of vitamins in the 1900s carrots became recognized as a rich source of β-carotene along with some protein, carbohydrates, fiber, and fat.

PCA for nutritional revealed new insights for the domestication of carrot. PCA divided selected 64 genotypes into two groups, Asia, and Europe. This finding is in agreement with the statements of Baranski et al. (77), Iorizzo et al. (78), and Grzebelus et al. (79) that cultivated germplasm could be divided into eastern and western gene pools and the carrot evolution was based on morphological markers (80). Moderate to high variations in morphological parameters were observed. There was wide range of core and cortex pigmentation found having red, yellow, orange, and white colors. These variations can be attributed to the genetic and environmental effects (81) and agrees the hypothesis by Arif et al. (82), carrot genotypes have the great potential of variability for yield relating traits (root traits) associated with its nutrition.

Correlation is considered as a helping tool in the selection of desirable traits for breeding programs. Nutritional parameters in this study displayed a range of genetic variation and similar trends were observed to measure genetic variability among diverse genotypes of carrot (83). The significant genotypic control of β-carotene accumulation observed in this research supports the findings of Buishand and Gabelman (84), Ellison (85), Sarker (58), and Simon (47), where regulatory genes are responsible for the biosynthesis and accumulation of β- carotenoids content and hence, breeding has doubled carotenoid levels for the last 60 years (47). Genotypic control of carbohydrates calories and vitamin C also contributes to improvement of the crop. Total sugar content ranged from 3 to 8%, in this study and both genotype and environment have been reported to influence sugar content in previous research (86, 87) so all these multiple factors must be monitored to improve carrot quality (88). The relative magnitude of vitamin C, moisture, crude fat, crude protein and carbohydrates for β-carotene synthesis observed with path analysis support reports by Cavagnaro (89) and Yadav et al. (90) where the authors explained that these factors also contribute to the enhancement of total dissolved solids and dry matter and also influences the processing quality of carrot.

Moisture content is an important factor of food before consumption. Moisture affects physical and chemical properties of food associated with shelf life. All genotypes had 70–80% moisture content except for several from Indian, North American and Europe, with a range between 10-88-90%. A negative correlation of vitamin C, carbohydrates and calories with moisture content is an important factor in controlling microbial growth and increasing shelf life of the products according to Chukwu and Abdullahi (91) who found that lower moisture is beneficial for storage and better shelf life.

Total soluble solids (TSS) include sugars, vitamins, and minerals. In the present study, the majority of genotypes had average to high TSS in carrots from in or near carrot's Central Asian region of domestication. TSS content increases during maturation of storage root and increased TSS content of carrots was also observed during storage period by Lingaiah and Huddar (92) and Jitender et al. (93). It was also observed that carrot packaging significantly affected the changes in TSS content of carrots during storage period (94). Low temperature carrot storage (1°C) maintained TSS content better than carrots stored at room temperature (92, 93, 95, 96).

In most of the genotypes, pH was slightly acidic as observed in carrot puree by Abbas and Khoudi (97) and Arqha (98) reported an average pH of the carrot between 4.9 and 5.2. Indeed, according to Anonyme (99), pH of some carrot may vary with varietal characteristics and growing conditions. The relationship between the slightly acidic nature of carrot and its organoleptic profile warrants additional study.

β-carotene exhibited higher values in fresh cut carrots than carrot products in this study because carrots were mixed with other colorless or lower carotene ingredients. This was especially noted in the carrot juice product which had much lower carotene content than other products. The low mineral content of the carrot juice product was also likely attributable to it mixture with low mineral ingredients. Vitamin C present in carrot-orange mix juice may also have lowered β-carotene content as was observed with the involvement of vitamin C in pineapple juice blended with orange and carrot which was attributed to reduced β-carotene during storage for 2 months (100). Carrot jam had the highest concentrations of β-carotene (6.5 mg/100 g) followed by carrot candies (4.8 mg/100 g) and carrot-orange mix juice (1.2 mg/100 g). This difference could be due to processing along with the addition of sugar, preservatives in jam, and oranges in the carrot juice. This significant increase in carrot jam agrees with Sant'Ana et al. (7) and Miglio et al. (101) where the authors reported the highest stability of β-carotene after boiling carrot for food preparation rather than any other cooking methods. In this context the boiling of carrots probably improved β-carotene in carrot jam.

Increased antioxidant capacity of carrot jam was also observed by Renna et al. (102), where they observed a 44% increase in antioxidant activity with yellow type carrot relative to other colors. No significant differences in antioxidant activity was observed among carrot products or among genotypes in this study but yellow carrots were not included. According to Sarker et al. (64) vegetables possessing high antioxidant quenching property can be consumed as nutraceutical products for nutrient deficient community.

While all products retained their nutrition for 3 months in this study candies stored at (±27°C started to degrade in the third month due to fungal attack. This storage temperature was used to reflect typical storage and handling practices of candies. This degradation is attributable to this high storage temperature, but the shelf life of carrot candies could be extended up to 6 months by storing at low temperature (1–3°C) (103). Kaur et al. (104) also observed carrot products treated with chemical additives improved physiochemical, phytochemical, antioxidant and shelf life while in this research carrot candies were only dipped into sugar syrup and no chemical treatment was applied. Based on these studies it is possible to stabilize carrot candies shelf-life, and with those studies either refrigerated storage or additives extend storage are recommended for long-sored carrot candies prepared by methods used in this study.

The studies by Sethi and Anand (103), Renna et al. (102), Kaur et al. (104), and Owolade et al. (100) noted above note a relatively few examples of carrot use for nutraceutical application. Given the extent of carrot crop production globally, the development of more long-storage products like jams, candies with preservatives, and “fruit leathers” would be valuable for global regions with minimal refrigerated long-storage. While carrot roots have relatively long post-harvest storage life than most fruit and vegetable crops, additional products could not only extend storage bust also increase overall consumption of this nutrient-rich crop.

## Conclusion

Correlation among various carrot attributes, particularly those for yield and nutritional contributing traits, is important for guiding future improvement of carrots. The wide range of variability recorded for various morphological and nutritional characters was important in identifying superior raw products genotypes in the development of nutritionally fortified nutraceutical products for this study. Among all the prepared products, carrot jam had the highest β-carotene and antioxidant content, while carrot candies also contained high levels of β-carotene and antioxidants and also demonstrated highest mineral and dry matter content. These results support the goal of this study in developing products attractive to consumers that can be used to alleviate vitamin A deficiency related disease. Consumption of these products can help in reducing xerophthalmia and other vision disorders like macular degeneration, increasing fertility, cognitive skills enhancement and providing protection against communicable and non-communicable diseases and oxidative stress. The incidence of these diseases is high in rural areas where no firsthand β-carotene supplements are available. This study proposed carrot-based products enriched in β-carotene and antioxidants that could be available to rural community at lower cost. Carrot jam and candies can be an alternative source to fulfill β-carotene requirement of 4 mg/100 g to 6.5 mg/100 g β-carotene per day along with significant antioxidant capacity which is in accordance with the recommended intake level by the USDA. Based on these studies, extended research is required to create additional high-quality products and to develop novel carrot hybrids which combine the blend of crop productivity with nutritional and functional attributes suitable for sustainably and locally produced nutraceutical products.

## Data Availability Statement

The datasets presented in this study can be found in online repositories. The names of the repository/repositories and accession number(s) can be found in the article/Supplementary Material.

## Author Contributions

NR: investigated, visualized, and wrote this original draft. ZYo: conceptualized, supervised, and helped in editing/reviewing. ZYa: post-harvest nutritional evaluation and carrot products development. MM: morphology data collection and statistical data analysis. AY, MR, AA, BS, and HY: review and edited. MN: crop cultivation. PS: conceptualized and assisted in editing/reviewing. All authors contributed to the article and approved the submitted version.

## Funding

This project was supported by Punjab Agricultural Research Board (PARB) under CGS grant No. 929, project “Phenotypic and Genotypic exploration of worldwide carrot germplasm to enhance its value-added applications in Pakistan”, and United States Department of Agriculture (USDA), Agricultural Research Service (ARS), under grant No. 5090-21000-069-00D, project, “Trait Discovery, Genetics, and Enhancement of Allium, Cucumis, and Daucus Germplasm”.

## Conflict of Interest

The authors declare that the research was conducted in the absence of any commercial or financial relationships that could be construed as a potential conflict of interest.

## Publisher's Note

All claims expressed in this article are solely those of the authors and do not necessarily represent those of their affiliated organizations, or those of the publisher, the editors and the reviewers. Any product that may be evaluated in this article, or claim that may be made by its manufacturer, is not guaranteed or endorsed by the publisher.
