# Supplementary material for: Development of Carrot Nutraceutical Products as an Alternative Supplement for the Prevention of Nutritional Diseases
Source: Front Nutr. 2022 Jan 3;8:787351. doi: 10.3389/fnut.2021.787351 (PMC8761950; doi:10.3389/fnut.2021.787351)
Supplement: Supplementary file 1 [file Table_1.DOC]

**Table S1: Root Based Phenotypic Plasticity of 64 *Daucus carota* L. Carrot Genotypes**

| **Genotype** | **Origin** | **Root Length (cm)** | **Root diameter (mm)** | **Root weight (kg)** | **Shoulder width (mm)** | **Core diameter (mm)** | **Root Shape** | **Root Tapering** | **Core color** | **Cortex color** | **Green Shoulder** | **Red shoulder** | **Lateral root Growth** |
| --- | --- | --- | --- | --- | --- | --- | --- | --- | --- | --- | --- | --- | --- |
| T29 | Pakistan | 27 | 41 | 0.27 | 40.85 | 18.85 | Tapering | Intermediate | White | Red | Absent | High | Medium |
| PI 218076 | Pakistan | 63 | 30 | 0.146 | 39 | 9.6 | Tapering | Intermediate | Red | Red | Medium | High | High |
| PI 269486 | Pakistan, Northern Areas | 24 | 30 | 0.13 | 37 | 19.26 | Tapering | Intermediate | Yellow | Yellow | High | Absent | High |
| PI 269487 | Pakistan | 22 | 40 | 0.25 | 48 | 15.96 | Tapering | Intermediate | Orange | Orange | Absent | High | High |
| Ames 22389 | Nepal | 28 | 36 | 0.38 | 42 | 5.77 | Tapering | Intermediate | Yellow | Red | Medium | Light | High |
| Ames 25024 | India | 26 | 50.15 | 0.235 | 55.8 | 3.5 | Tapering | Intermediate | Yellow | Red | Medium | Medium | Medium |
| Ames 25042 | India | 28 | 45 | 0.42 | 45 | 15.39 | Tapering | Intermediate | Red | Red | Absent | High | Medium |
| Ames 25043 | India | 29 | 38 | 0.33 | 68 | 12.45 | Tapering | Intermediate | Yellow | Red | High | Light | Medium |
| PI 163239 | India, Delhi | 34 | 35 | 0.2 | 52 | 7.34 | Tapering | Intermediate | White | Yellow | Absent | High | High |
| PI 163241 | India Punjab | 30 | 31 | 0.12 | 42 | 10.73 | Tapering | Intermediate | Yellow | Yellow | High | Light | High |
| PI 164136 | India Madhya Pradesh | 25.6 | 26 | 0.14 | 48 | 12.01 | Tapering | Intermediate | Yellow | Red | Light | High | High |
| PI 164344 | India, Tamil Nadu | 25 | 18 | 0.035 | 23 | 9.95 | Tapering | Intermediate | Orange | Orange | Medium | High | High |
| PI 164798 | India, Maharashtra | 24 | 21 | 0.075 | 44 | 10.18 | Tapering | Intermediate | Yellow | Red | Light | Light | High |
| PI 174828 | India, Uttar Pradesh | 27 | 46 | 0.265 | 58 | 8.15 | Tapering | Intermediate | Yellow | Red | Light | High | High |
| PI 175132 | India, Uttar Pradesh | 23 | 18 | 0.14 | 49 | 7.52 | Tapering | Intermediate | Yellow | Orange | Light | High | High |
| PI 179690 | India, Gujrat | 31 | 36 | 0.305 | 66 | 6.2 | Tapering | Intermediate | Yellow | Orange | Medium | High | Medium |
| PI 180436 | India, Gujrat | 19 | 26 | 0.13 | 41 | 5.96 | Tapering | Intermediate | Yellow | Red | Medium | Medium | High |
| PI 271044 | India | 26 | 21 | 0.08 | 35 | 12.9 | Tapering | Intermediate | White | White | Absent | High | Medium |
| PI 271473 | India, Rajasthan | 25 | 24 | 0.115 | 37 | 21.62 | Tapering | Intermediate | White | White | Absent | High | High |
| PI 288765 | India, Gujrat | 25 | 30 | 0.18 | 52 | 14.13 | Tapering | Intermediate | Yellow | Yellow | Medium | Medium | High |
| PI 652253 | India, Uttar Pradesh | 24 | 38 | 0.24 | 50 | 13.66 | Tapering | Intermediate | Red | Red | Medium | Medium | Light |
| PI 652255 | India, Uttar Pradesh | 18 | 23 | 0.075 | 36 | 8 | Tapering | Intermediate | Orange | Orange | High | Absent | Light |
| PI 652259 | India | 23 | 31 | 0.1 | 41 | 16.54 | Tapering | Intermediate | Yellow | Orange | Medium | Medium | Medium |
| PI 652260 | India, Haryana | 29 | 40 | 0.027 | 49 | 8.15 | Tapering | Intermediate | Yellow | Red | Absent | Medium | Medium |
| PI 652261 | India, Haryana | 25 | 45 | 0.25 | 40 | 16.54 | Tapering | Intermediate | Yellow | Red | Medium | Medium | Medium |
| PI 652410 | India, Rajasthan | 26 | 24.5 | 0.118 | 37.5 | 10.17 | Tapering | Intermediate | Yellow | Red | Medium | Medium | Medium |
| PI 164942 | Turkey Istanbul | 28 | 30 | 0.148 | 49 | 10.02 | Tapering | Intermediate | Yellow | Red | Light | Medium | Medium |
| PI 167211 | Turkley, Icel | 34 | 21 | 0.095 | 31 | 25.73 | Tapering | Intermediate | Red | Red | Absent | High | Medium |
| PI 169483 | Turkey, Izmir | 23 | 18 | 0.06 | 25 | 5.34 | Tapering | Intermediate | Yellow | Yellow | Absent | High | Medium |
| PI 169485 | Turkey, Istanbul | 26 | 33 | 0.175 | 47 | 11.48 | Tapering | Intermediate | Yellow | Orange | High | Light | Medium |
| PI 169486 | Turkey, Kirklareli | 22 | 18 | 0.35 | 20 | 8.92 | Tapering | Intermediate | Yellow | Yellow | High | Light | Medium |
| PI 175718 | Turkey, Kayseri | 20 | 10 | 0.025 | 25 | 9.28 | Tapering | Intermediate | Yellow | Red | Light | High | Medium |
| PI 652356 | Turkey, Aydin | 22 | 39 | 0.25 | 54 | 10.75 | Tapering | Intermediate | Yellow | Red | Light | Medium | Medium |
| PI 652400 | Turkey, Denizli | 27 | 19.4 | 0.375 | 52 | 9.98 | Tapering | Intermediate | Yellow | Red | Light | Absent | Medium |
| PI 652404 | Turkey, Denizli | 25 | 31 | 0.165 | 39 | 7.79 | Tapering | Intermediate | Yellow | Red | Light | Absent | Medium |
| PI 177383 | Syria | 50.5 | 19.6 | 0.26 | 31.5 | 10.22 | Tapering | Intermediate | Yellow | Orange | Light | Absent | Light |
| PI 223362 | Iran | 20 | 42 | 0.22 | 44.8 | 15 | Tapering | Intermediate | White | White | Absent | High | Medium |
| PI 211590 | Afghanistan, Badakhshan | 25 | 36.3 | 0.155 | 44.7 | 22.6 | Tapering | Intermediate | White | White | Absent | High | Medium |
| PI 219914 | Afghanistan, Kabul | 21 | 21 | 0.065 | 34 | 3.73 | Tapering | Intermediate | Red | Red | High | High | High |
| PI 220014 | Afganistan, Kabul | 29 | 20 | 0.06 | 29 | 3.6 | Tapering | Intermediate | Yellow | Yellow | Absent | High | High |
| PI 220517 | Afganistan, Balkh | 21 | 36 | 0.185 | 57 | 3.92 | Tapering | Intermediate | Yellow | Yellow | Absent | High | High |
| PI 220657 | Afghanistan, Herat | 19.1 | 17 | 0.07 | 40 | 13.19 | Tapering | Intermediate | Yellow | Yellow | Medium | Medium | Absent |
| PI 220794 | Afghanistan, Ghazni | 33 | 24 | 0.175 | 43 | 5.35 | Tapering | Intermediate | White | Orange | High | Light | Absent |
| PI 220795 | Afganistan, Kondoz | 34 | 29 | 0.155 | 44 | 11.65 | Tapering | Intermediate | Red | Red | High | Medium | Medium |
| PI 223504 | Afghanistan, Nangarhar | 19 | 36 | 0.255 | 56 | 13.18 | Tapering | Intermediate | Yellow | Yellow | Absent | High | High |
| PI 223777 | Afghanistan, Badakhshan | 22 | 38 | 0.25 | 41.5 | 19.91 | Tapering | Intermediate | Yellow | Yellow | Absent | High | High |
| PI 418967 | China, Shaanxi | 20.5 | 12 | 0.22 | 20 | 20 | Tapering | Intermediate | Yellow | Yellow | Absent | High | High |
| PI 193506 | Ethiopia | 52 | 30 | 0.11 | 34 | 3.78 | Tapering | Intermediate | Yellow | Orange | Medium | Light | Light |
| Ames 32040 | Tunisia | 21 | 25 | 0.145 | 43 | 14.55 | Tapering | Intermediate | Yellow | Red | Absent | High | Medium |
| PI 288242 | Egypt | 28 | 39 | 0.299 | 54 | 22.43 | Tapering | Intermediate | W | W | Absent | High | Light |
| PI 279776 | Egypt, Giza | 27 | 9.65 | 0.115 | 35.05 | 16.54 | Tapering | Slight | Yellow | Yellow | Medium | High | Absent |
| NSL 6187 | United States, California | 38 | 40 | 0.21 | 35 | 13.02 | Tapering | Intermediate | Yellow | Orange | High | Absent | Medium |
| NSL 26503 | United states Michigan | 23 | 11 | 0.015 | 11 | 5.18 | Tapering | Slight | White | White | Absent | Absent | High |
| NSL 199861 | United states United states WI | 68 | 24 | 0.8 | 41 | 8.83 | Tapering | Intermediate | Orange | Orange | Absent | High | Medium |
| PI 632388 | United States, Illinois | 24 | 38 | 0.23 | 39 | 11.34 | Tapering | Intermediate | Yellow | Red | High | Light | High |
| PI 632392 | United States, Wyoming | 17 | 25.2 | 0.185 | 29.5 | 12.11 | Tapering | Intermediate | Yellow | Red | High | Light | Light |
| PI 665468 | United States, California | 24 | 13.7 | 0.27 | 51.45 | 13.5 | Tapering | Acute | Yellow | Red | Medium | Medium | High |
| PI 226309 | Mexico, Federal District | 20 | 22 | 0.125 | 29.3 | 10.24 | Tapering | Intermediate | Yellow | Orange | Absent | High | High |
| PI 325984 | Russian Federation | 30 | 31.6 | 0.15 | 41.3 | 14 | Tapering | Intermediate | Yellow | Orange | Absent | High | Light |
| PI 325998 | Ukraine | 19 | 23 | 0.055 | 36 | 18.01 | Tapering | Intermediate | Yellow | Orange | Medium | Absent | Light |
| PI 379327 | Serbia | 25 | 20.17 | 0.09 | 33.7 | 9.3 | Tapering | Slight | Yellow | Yellow | Medium | Absent | Light |
| PI 379328 | Serbia | 26 | 26.5 | 0.12 | 36 | 19.81 | Tapering | Intermediate | White | Orange | Absent | Absent | High |
| PI 634658 | France | 25 | 48 | 0.305 | 52 | 7.25 | Tapering | Intermediate | Yellow | Orange | Medium | Medium | Light |
| PI 652169 | France | 25 | 15.95 | 0.2 | 39.8 | 7.20 | Tapering | Intermediate | Yellow | Red | Medium | Absent | Light |
